# A mixed methods evaluation of the acceptability of therapy using LEGO® bricks (LEGO® based therapy) in mainstream primary and secondary education

**DOI:** 10.1002/aur.2725

**Published:** 2022-04-09

**Authors:** Amy Barr, Elizabeth Coates, Ellen Kingsley, Gina Gomez de la Cuesta, Katie Biggs, Ann Le Couteur, Barry Wright

**Affiliations:** ^1^ Clinical Trials Research Unit, School of Health and Related Research The University of Sheffield Sheffield UK; ^2^ COMIC Leeds and York Partnership NHS Foundation Trust Leeds UK; ^3^ Play Included Community Interest Company Cambridge UK; ^4^ Population Health Sciences Institute Newcastle University Newcastle upon Tyne UK; ^5^ Health Sciences University of York York UK

**Keywords:** autism, LEGO®, play brick therapy, qualitative research, questionnaires, social communication

## Abstract

Many autistic children and young people need extra support with social skills. Social skills programmes, such as LEGO® based therapy (LBT), are commonly used to help with these difficulties. The aim of this study was to examine the acceptability of LBT using qualitative interviews and questionnaires with facilitators and parents/guardians on behalf of autistic children and young people. Acceptability was measured in line with constructs of the Theoretical Framework of Acceptability. Questionnaires were analyzed descriptively and between group comparisons were undertaken using the Mann–Whitney *U* Test. Telephone interviews were undertaken with a sub‐sample of facilitators. All interviews were recorded, transcribed verbatim, and framework analysis was performed by two researchers supported by NVivo. The questionnaire response rate was 80% for facilitators and 77% for parents/guardians. Overall acceptability, measured on a 1–5 (minimum–maximum) scale, was high for both facilitators and parents/guardians with a median (range) of 5 (4–5) and 4 (3–5), respectively. Facilitators rated the acceptability of the programme significantly higher overall than parents (*p* < 0.001). Facilitators reported that participants and wider school staff viewed the programme positively. They observed improvements in communication and social skills during the sessions. Potential barriers to programme delivery, such as resources and staff schedules, were identified but facilitators reported that these challenges did not outweigh the benefits. There is increasing emphasis on the role of schools in seeking to improve social outcomes for autistic children therefore this high degree of acceptability makes this an attractive school‐based programme for schools, autistic children and their families.

**Lay Summary:**

Social skills programmes, such as LEGO® based therapy (LBT), are often used to help autistic children and young people with their social skills. The acceptability of LBT with school staff and parents/guardians on behalf of children and young people was explored using interviews and questionnaires. Our results show that LBT is viewed as a highly acceptable programme that can help autistic children and young people improve their communication and social skills.

## INTRODUCTION

Autism is a lifelong neurodevelopmental condition which is estimated to affect 1.6% of children and young people in the UK (Baird et al., 2006). Many autistic children and young people need extra support with social skills, often finding it hard to form and maintain friendships (American Psychiatric Association, 2013). The condition is also characterized by sensory reactivities and stereotyped behaviors. Social skills programmes are commonly used to help with these difficulties, however they tend to be adult‐led in nature and use a skills deficit model which may limit their overall success (Gates et al., 2017; Howlin et al., 2004).

LEGO® based therapy (LBT) (LeGoff et al., 2014) is a group social skills programme designed for children and young people with social communication difficulties including ASD. It has become increasingly popular in the UK despite limited research to date (North Yorkshire County Council Intervention Guidance, 2019). Its aim is to use collaborative building of LEGO sets between small groups of autistic children and young people to provide positive social opportunities and support social development. Autistic children and young people work collaboratively in groups to build LEGO sets following instructions or designing their own creations as a team. The programme was created by Dr Daniel LeGoff who showed in early studies that it may be helpful for children and young people who need extra support for social and communication skills and more supportive opportunities to be with others and make friends (LeGoff, 2004; LeGoff & Sherman, 2006).

To date only one small randomized controlled trial (RCT) has been undertaken to investigate the effect of LBT (Owens et al., 2008). Results were promising, however the sample size was small (*n* = 47) and full randomization was not used. A scoping review of LBT carried out in 2017 (Lindsay et al., 2017) concluded from the 15 studies included that more rigorously designed evaluation of the programme with larger samples, randomization processes, and standardized measures was necessary. One recent small‐scale study was undertaken in a school context in 2020, although this was not an RCT and included only six participants (Levy & Dunsmuir, 2020). There was a positive effect on the frequency of social initiations and responses, duration of social engagement and positive social behaviors for five of the six participants. Some evidence of generalization of these skills at home or other school settings was observed, albeit not consistently.

As noted in the 2017 scoping review (Lindsay et al., 2017), studies of LBT to date have lacked methodological consistency, power, standardized measures, and full randomization, nor have they assessed cost‐effectiveness or acceptability of the intervention. Acceptability can be defined as ‘a multi‐faceted construct that reflects the extent to which people delivering or receiving a healthcare intervention consider it to be appropriate, based on anticipated or experienced cognitive and emotional responses to the intervention’ (Sekhon et al., 2017: 4). Understanding the acceptability of a healthcare intervention is key to successful implementation as it may influence uptake by trainers, therapists and participants as well as impact both recruitment and retention of any planned intervention or evaluation. Healthcare interventions are often multi‐faceted and are delivered across healthcare settings and staff groups (Craig et al., 2008), therefore, successful implementation of an intervention depends on the acceptability of both intervention deliverers and recipients (Diepeveen et al., 2013).

Embedded in the I‐SOCIALISE trial, a fully powered pragmatic cluster RCT evaluating the clinical and cost‐utility of LBT for autistic children and young people in a school environment, this study aimed to examine the acceptability of the intervention (referred to as ‘the programme’ throughout) using qualitative interviews and questionnaires with both parents and facilitators (teachers or teaching assistants trained to deliver the programme). For full details of the I‐SOCIALISE RCT, see Varley et al. (2019).

## METHODS

### Research design and setting

This was a mixed methods evaluation of the acceptability of LBT embedded within the I‐SOCIALISE RCT undertaken in mainstream primary and secondary schools in three areas in the North of England.

### Participants and eligibility

This study was part of the I‐SOCIALISE trial, and the overall eligibility criteria for children and young people, parents/guardians and teachers/teaching assistants have previously been reported – see Varley et al. (2019) and supplementary material S1.

All parents/guardians of autistic children and young people that met the I‐SOCIALISE trial eligibility criteria, and were randomized to receive the programme, were asked to complete a questionnaire to assess the acceptability of LBT as a part of the trial follow up at 20 weeks post‐randomization. Facilitators (usually teachers or teaching assistants [TA] who were trained to deliver LBT in those schools randomized to undertake the programme) were also asked to complete the questionnaire to assess acceptability at 20‐weeks post‐randomization.

Qualitative interviews were carried out with a sub‐sample of facilitators who had, at the beginning of the study, consented to further contact regarding taking part in a telephone interview (*n* = 59). Participants were purposively sampled across location and school type (primary and secondary). The purpose of the interviews was to understand facilitator experiences of delivering and implementing the programme, as well as to explore acceptability, both to them, amongst school staff, and by proxy, acceptability to parents and autistic children and young people. Data collection ceased at data saturation (*n* = 16).

### Intervention

LBT is a group‐based social skills programme designed for use with children and young people on the autism spectrum. It allows children and young people to learn and practice their communication and social skills whilst building LEGO models with their peers in a structured format, with oversight from a trained facilitator. Collaborative building between group members is a fundamental aspect of LBT. Participants are asked to build a LEGO model together, taking turns in the roles ‘builder’, ‘supplier’, and ‘engineer’ as they work their way through a set of pre‐written instructions, or build their own creation as a team. A key aim of LBT is to allow autistic children to learn through play and develop their social and communication skills in a fun and familiar setting. Trained facilitators guide participants when social or practical issues arise as they play in the group, as well as allowing the participants to take the lead, wherever possible during the sessions. Children and young people develop their own set of behavioral rules for the group and can receive rewards during the sessions for working together, positive social behaviors and skills in LEGO building. LEGO can also provide children and young people with a tool to form friendships.

As part of the I‐SOCIALISE study, LBT was run only in those schools randomized to undertake the programme. Following randomization, facilitators underwent face‐to‐face training and were given training materials to guide them during the sessions. All sessions were delivered in person and to groups of three participants. The groups included autistic children and young people together with, in some schools, other neurotypical children and young people chosen by the schools. Schools were encouraged to use, wherever possible, the same quiet room for the LBT sessions to keep a consistent environment, with few disturbances, and for storage of the materials. Schools were asked to run 12 weekly 1‐h sessions. It was recognized that on occasions (such as due to illness, holidays, and other timing issues) it might be necessary for schools to plan more than one session per week.

### Ethics

Ethical opinion for this study was obtained from the University of York Research Ethics Committee (17/03/2017), and governance approval was granted by the Health Research Authority (18/HRA/0101). All participants (parents and facilitators) provided written informed consent to complete a questionnaire and a sub‐set of facilitators gave consent to take part in the audio‐recorded interviews. All child and young person participants gave assent to take part in the trial.

### Data collection

A questionnaire based upon the Theoretical Framework of Acceptability (TFA) (Sekhon et al., 2017) was designed by an expert group in the study team, which included PPI representatives, to assess the acceptability of the programme for parents/guardians and facilitators. Before finalization, the questionnaire was piloted by the parent representative members of the I‐SOCIALISE trial management group (TMG), and two teaching staff with prior experience of delivering LBT who were not involved in the trial. Acceptability was measured across the constructs of the TFA using a 5‐point Likert scale from Strongly Disagree (1) through to Strongly Agree (5). A free text response box was also included for parents/guardians to add additional information on each questionnaire. For more information on the questionnaire, please refer to Supplementary materials S2 and S3.

Facilitator and Parent/guardian questionnaire data were collected as part of the I‐SOCIALISE trial follow‐up at 20 weeks post‐randomization at a face‐to‐face visit, via post, or via an online questionnaire.

Qualitative interviews were conducted with facilitators upon completion of programme delivery. The Theoretical Framework of Acceptability (Sekhon et al., 2017) was used to aid the design of the interview schedule, and to guide data analysis, helping to understand the acceptability of LBT for facilitators, as well as the acceptability amongst parents/guardians and on behalf of their children.

The Theoretical Framework of Acceptability is made up of seven component constructs (from figure 3 in Sekhon et al., 2017):

- ‘affective attitude: how an individual feels about the intervention;
- burden: the perceived amount of effort that is required to participate in the intervention;
- ethicality: the extent to which the intervention has a good fit with the individual's value system);
- intervention coherence: the extent to which the participant understands the intervention and how it works;
- opportunity costs: the extent to which benefits, profits or values must be given up to engage in the intervention;
- perceived effectiveness: the extent to which the intervention is perceived as likely to achieve its purpose; and
- self‐efficacy: the participant's confidence that they can perform the behavior(s) required to participate in the intervention.’

The interview schedule was piloted with two staff members from Local Authorities who had prior experience of implementing LBT, before finalization. A copy of the interview schedule is given in Supplementary material S4.

Facilitators who had consented to be contacted were invited to participate via an invitation email. The telephone interviews were conducted by a postgraduate researcher (AB). All interviews were audio recorded and transcribed verbatim.

### Data analysis

Questionnaire data from parents and facilitators were analyzed descriptively. Between group comparisons were undertaken using the Mann–Whitney *U* Test, due to the skewness of the data. Open ended responses were subjected to a simple content analysis.

Interview data were analyzed using the Framework analysis approach (Spencer et al., 2003), aided by NVivo software (version 12). The Framework analysis approach is well established in applied health services research and is not aligned with any particular epistemological standpoint and therefore provided a good fit for this research (Gale et al., 2013). Interview transcripts were coded by two independent members of the research team (AB and LC). Coders met regularly to create a coding framework and to verify the grounding of all codes in the original interview data. The coding framework was revised during interview analysis to permit the inclusion of new codes, and ensure codes found to be redundant were removed. Upon completion, the coding framework was reviewed by members of the I‐SOCIALISE TMG and Trial Steering Committee to confirm validity, coherence, and relevance.

## RESULTS

A total of 98 schools took part in the I‐SOCIALISE study. Of these, 50 schools were allocated to undertake the programme: 127 children and young people took part in LBT, and 81 facilitators delivered the sessions. In total, 98/127 (77%) parents/guardians and 65/81 (80%) facilitators returned acceptability questionnaire data.

Qualitative interviews were undertaken with 16 facilitators, 12 from primary schools. The median (range) duration of the interviews was 40 min (22–61 min). A total of nine participants were from schools in Leeds, two were from schools in Sheffield and one was from a school in York. At the time of interview, 10 participants worked as TAs, four as Special Educational Needs Coordinators (SENCos) and two as teachers. All participants had previous experience with autistic children and young people or other SEN experience, and nine specified they had prior experience, or were familiar with, LBT.

Demographic information for all participants is shown in Table 1.

**TABLE 1:** Demographic characteristics of facilitators and parents/guardians

	Survey respondents		Interview participants
Characteristic	Parents/guardians (*n* = 98)	Facilitators (*n* = 65)	Facilitators (*n* = 16)
Median age [range]	38 [28–54]	43 [20–62]	48 [≤25–≥55^b^]
Gender			
Female	87 (89%)	55 (85%)	14 (88%)
Male	8 (8%)	9 (14%)	2 (13%)
Not specified	3 (3%)	1 (2%)	0 (0%)
Ethnicity *N* (%)			
English/Welsh/Scottish/	81 (83%)	60 (92%)	≥80%^b^
Northern Irish/British			
Other ethnic group	16 (16%)	5 (8%)	^b^
Prefer not to say	1 (1%)	0 (0%)	^b^
Relationship to child *N* (%)			
Mother/father	97 (99%)		
Other person with parental responsibility	1 (1%)		
Role *N* (%)			
Teaching assistant		41 (63%)	10 (63%)
Other^a^		24 (37%)	6 (37%)
Median years' of experience [range]		11 [1–39]	14 [1–25]

^a^ Includes: Teacher, learning mentor and SENCO.

^b^ Approximation and/or suppression due to low numbers.

### Questionnaire findings

Table 2 shows an overview of the acceptability of LBT for facilitators, and parents/guardians of autistic children and young people (rating acceptability on behalf of their children). Overall acceptability, as measured on a 1–5 (minimum–maximum) scale, was high for both facilitators and parents/guardians with a median (range) of 5 (4–5) and 4 (3–5), respectively. Facilitators rated the acceptability of the programme higher overall than parents, and this difference was statistically significant (*p* < 0.001).

**TABLE 2:** Acceptability of LEGO®‐based therapy intervention to facilitators and parents/guardians – All participants

	Facilitators (*n* = 65)^a^			Facilitators (*n* = 65)^c^	
Acceptability construct	Median (range)	Parents (*n* = 98)^a^	*p* value^b^	% reporting 4 or 5	Parents (*n* = 98)^c^
Affective attitude	5 (4–5)	5 (4–5)		100	91.8
Burden	5 (4–5)	3 (2.75–4)		86.2	49.0
Ethicality	4 (4–5)	4 (4–5)		93.9	78.4
Intervention coherence	5 (5–5)	3 (3–4)		93.9	46.8
Opportunity costs	5 (4–5)	4 (4–5)		95.4	83.5
Perceived effectiveness – General	4 (4–5)	4 (3–5)		92.2	69.1
Perceived effectiveness – Social skills	4 (4–5)	4 (3–4)		90.6	66.0
Perceived effectiveness – Academic confidence	5 (4–5)	4 (4–5)		89.2	75.3
Perceived effectiveness – Communication skills	5 (4–5)	4 (4–5)		95.4	84.5
Perceived effectiveness – Behavior	4 (4–5)	4 (3–4)		73.4	54.6
Perceived effectiveness – Overall	5 (4–5)	4 (3–5)		87.7	69.9
Self‐efficacy	5 (4–5)	4 (4–5)		100	79.6
Overall acceptability	5 (4–5)	4 (3–5)	<0.001	91.6	70.8

^a^ Median (range).

^b^ Mann Whitney *U* Test.

^c^ % rating intervention positively (pooled Likert scores 4 and 5). Higher scores indicate a greater level of acceptability.

Facilitators rated all individual constructs of the TFA positively, with median ratings of 5 or 4. All of the facilitators stated that they liked the programme (affective attitude) and felt confident delivering LBT (self‐efficacy). All other constructs received positive ratings (i.e., percentage of participants scoring 4 or 5), with the lowest score of 73% (perceived effectiveness on behavior) still being high. Overall, 92% of facilitators rated the acceptability of LBT positively.

For parents/guardians, ‘affective attitude’ was the only TFA construct given the maximum median score of 5, with 92% of parents/guardians assessing LBT positively. The individual TFA constructs rated least positively by parents/guardians were ‘intervention coherence’ (47%), ‘burden’ (49%) and ‘perceived effectiveness on behavior’ (55%). The overall acceptability score for parents/guardians was lower than facilitators at 71.

Tables 3 and 4 show a summary of the acceptability questionnaire open‐ended responses given by facilitators and parents/guardians, respectively. In general, the facilitators reported that running LBT was a positive and rewarding experience, both personally, and in their opinion, for the children in their groups. They noted a number of perceived benefits of the programme, including improvement in children and young people's communication, social skills and confidence. Some facilitators said that these skills were not translated to a classroom setting. Facilitators also identified certain challenges during programme delivery, such as conflict between participants, and participants becoming fatigued as the sessions progressed. Five facilitators reported that their schools had continued LBT post‐trial and are offering it to other pupils.

**TABLE 3:** Summary of open‐ended qualitative responses on acceptability questionnaire – Facilitators (*n* = 45)

		Summary
1. Enjoyed LEGO®‐based therapy	1.1 Children and young people	Fifteen facilitators said that the children and young people involved in LEGO®‐based therapy enjoyed their sessions and looked forward to them every week
	1.2 Interventionist	Twenty‐two of the facilitators said that, despite certain challenges, LEGO®‐based therapy was a rewarding and useful intervention to run, and that they enjoyed delivering LBT sessions in their schools
2. Benefits	2.1 During sessions	Eighteen facilitators said that LEGO®‐based therapy has clear benefits, and they have seen improvements in communication, social skills, & confidence during the sessions. Three facilitators did say that, although they had seen the benefits during the sessions, these might not be reflected in the classroom
	2.2 Wider benefits	Eight of the facilitators said that their children and young people had been more confident and had improved communication in the wider school setting. Two facilitators also said that they had seen a positive effect in children and young people without ASD, including those with challenging behaviors
3. Resources		Two facilitators stated that they had trouble finding a suitable space for some of their sessions, as space in their schools was lacking. Two interventionist said that LEGO®‐based therapy was easy to set up and deliver
4. Challenges		Five of the facilitators found LEGO®‐based therapy challenging to run at times. One interventionist said that having two children and young people with ASD in the group was tough, as they needed a lot of prompting to communicate, whereas others found that they struggled to find a suitable space to hold the sessions. One interventionist said that their children and young people struggled with finding the necessary language to describe the pieces, and another said that their children and young people lost motivation after seven or eight sessions, making the final sessions more difficult to run
5. Implementation		Five facilitators said that their school has decided to continue with LEGO®‐based therapy, and are trialing it with more groups, or that they have rolled it out across their whole school. One school said they will be implementing this over a half term (6 weeks), then revisiting at a later date, rather than delivering it as a 12 weeks block
6. Recommendations		One interventionist said that her group would have liked to have done more than 12 sessions, however, another said that 12 weeks was too long, and that they would possibly split this up into two blocks in the future. Three secondary school facilitators said that LEGO®‐based therapy may be more suitable in a primary setting rather than secondary, and that secondary schools needed more complex sets for it to be effective. One of the facilitators said that they would include more free‐style in future groups, to allow children and young people to be more creative, and another said that they would only use children and young people in the same year group in the future

Abbreviation: LBT, LEGO® based therapy.

**TABLE 4:** Summary of open‐ended qualitative responses on acceptability questionnaire – Parents/guardians (*n* = 44)

		Summary
1. Enjoyed LEGO®‐based therapy		Twenty‐seven parents/guardians stated that their children and young people enjoyed taking part in LEGO®‐based therapy. Eleven parents/guardians said they had seen noticeable changes in their children and young people's behavior, communication, and social skills, however, seven parents/guardians said that, although their children and young people enjoyed the session, they did not see any impact at home. One parent suggested this may be because it is ‘too early’ to see any significant changes
2. Benefits	2.1 Social skills	Seven parents/guardians felt that LEGO®‐based therapy has improved their children and young people's social skills, as it helped them with making new friends and interacting with other children and young people in class
	2.2 Communication	Eight parents/guardians noticed that LEGO®‐based therapy had a positive impact on their children and young people's communication with their peers. Two parents/guardians also said that their children and young people has been more open and spoken about their feelings more at home
	2.3 Confidence	Two parents/guardians noticed that their children and young people had grown in confidence after taking part in LEGO®‐based therapy sessions
	2.4 Calmness	One parent stated that there is more relaxed and calmer since taking part in LEGO®‐based therapy
3. Children and young people/parent would like to continue LEGO®‐based therapy		Three parents/guardians would have liked their children and young people to continue with LEGO®‐based therapy because they have enjoyed it so much, and to continue to develop their skills further and apply these at home
4. Feedback from school		Four parents/guardians found that they were not sure how their children and young people got on during LEGO®‐based therapy, as they received little or no feedback from the school
5. Didn't enjoy LEGO®‐based therapy		Three of the parents/guardians stated that their children and young people did not enjoy LEGO®‐based therapy due to having lots of previous building experience, or wanting more structure to the sessions

Most parents/guardians reported that their children enjoyed being involved in LBT. As with facilitators, many parents noted that they had seen improvements in behaviors, communication, confidence and social skills, although, some explained that these improvements were not observed at home. A small proportion of parents/guardians reported that their children had not enjoyed LBT due to having previous experience of building more complex sets at home, making it hard for them wait for instructions instead of rushing ahead. Although all parents/guardians provided consent for their children to take part in the programme, some indicated that they were unaware of their child's experience—either from the child themselves or from the school staff.

### Interview findings

After familiarization with the transcribed interview data, initial codes were generated (Spencer et al., 2003). Following multiple reviews of the complete set of transcriptions by the two reviewers, the finalized framework contained 26 codes, across eight categories (see Supplementary material S5). The dataset was then indexed, and charts were created to summarize the content of each category, code (and sub‐code). To aid interpretation, the data were then mapped to the Theoretical Framework of Acceptability constructs (Sekhon et al., 2017) (see Supplementary material S6).

#### Theoretical framework of acceptability constructs

##### Affective attitude

Most facilitator participants viewed LBT positively. They explained that it was enjoyable to deliver and easy to explain to participants as the materials were familiar and an interesting resource to children and young people. Most also said the participants in their groups had enjoyed taking part in LBT, with some asking to continue with this after the study completed. A secondary school facilitator noted that the children and young people found the sessions to be a ‘light relief’ when compared to their usual timetables.

I think what made it fairly easy to talk to them about it, is because they all had a knowledge and an interest in LEGO prior to the programme. (Primary facilitator)

Overall, facilitators reported that the programme was successful, but not without challenges. For example, some of the participants already confident playing with LEGO found the roles and rules of the LBT group activities challenging, preferring to rush ahead to build, or became frustrated with their peers. In contrast, some facilitators noted that, for younger participants, certain LEGO sets were more difficult for those with fewer fine motor skills.

##### Burden

Many facilitators indicated that LBT was compatible with current working practices in their schools. They noted that having weekly protected time and a designated workspace made integrating the programme easier. Some explained the importance of using the same room to conduct LBT, and the challenge schools with limited space might face. Several noticed that sessions held in unfamiliar locations had been disruptive and upsetting for the participants.

Despite the overall positivity, most facilitators stated the greatest burden was scheduling weekly sessions that suited everyone's timetable. Some school staff were required to alter their timetables to fit in with LBT, and some children missed timetabled lessons. However, for most this was not considered to be unmanageable.

The class teacher is one member of staff down, so yes, it is, it is quite an impact and it's hard to maintain an intervention every single week without a break in a school because schools are fluid places and they are flexible and things happen. (Primary facilitator)

##### Ethicality

All facilitators agreed that LBT fitted in well with what they believe helps autistic children and young people. The majority stated that the structured nature (in terms of timing, venue and also the semi‐structured play activities) of the programme suits the needs of autistic children and young people, helping them to socialize and communicate. Many facilitators also described how LBT fitted with the goals of their schools, and supported their aims to be inclusive of all children and young people, including those with autism and other SEN.

I think (it fits in with school values) because we're just striving to be a fully inclusive school and to give kids on the SEND [Special Education Needs and Disabilities] register just the greatest opportunity they can. (Primary facilitator)

##### Intervention coherence

All facilitators stated they understood how to deliver LBT. Most noted that other teachers in their schools not involved in programme delivery struggled to fully understand the purpose and structured nature of LBT. To help combat this and improve wider understanding, staff members were often invited to observe sessions, and/or the facilitators had provided more detailed explanations in staff meetings.

It's been more of a problem to explain to staff, they think it's just an hour of playing with LEGO. And whereas, I had to explain how it works and whilst we're playing with LEGO for the hour, there was very much a structure to it. (Primary facilitator)

Despite the enthusiasm shown by most participants, a number of facilitators thought that the children and young people were not fully aware of the purpose of LBT and stated the importance of giving them a clear explanation about the structured nature and the roles within the play.

…but it's trying to talk to them about ‘it's not just building, we're not just playing, we're going to do it in a structured way and you'll each have a role to play’. So, once they get their head around that, then it's pretty much plain sailing from there. (Primary facilitator)

Facilitators in primary schools felt that parents had a good overall understanding but needed further explanation that their children were doing ‘more than just building’. They highlighted that ‘therapy’ may give parents a negative impression, and that terminology was important when discussing the programme, as was explaining to parents why their children were taking part. Secondary school facilitators stated they had had no feedback from the parents/guardians.

##### Opportunity costs

Most facilitators felt that the perceived benefits of LBT far outweighed any negative implications on resources or other opportunity costs (e.g., staff time, planning, etc.). As mentioned above, facilitators explained that the main issue was consistently scheduling weekly sessions.

They also noted that continuing LBT in their schools would incur a substantial financial cost for the LEGO sets, and that the benefits of the programme must balance this out to enable investment. Some schools had acquired sets through donations or had fundraising plans. However, one school had already identified this cost as a barrier to implementing LBT at their school in the future.

The other cost would be and that would be the real sticking point is, you know, having to buy the LEGO kits. That would have to be thought through carefully. (Primary facilitator)

Some facilitators also noted the opportunity costs for children, as they would miss academic lessons to participate in LBT sessions. However, efforts were made to schedule the sessions so that the children and young people would not miss core lessons, such as maths and English, to reduce the impact on their overall education.

##### Perceived effectiveness

All facilitators stated that they had seen benefits when running the programme, especially improvements in communication and socializing skills. Other reported benefits included team working, improved language skills, resilience during problem solving and increased confidence when addressing their peers. Some also saw friendships develop between group members that they felt would not have happened without LBT.

One boy is autistic, and the other boy that shows lots of autistic traits, both on the playground would basically just play by themselves or walk around the perimeter of the playground and just play on their own. And they are now best friends. (Secondary facilitator)

Most facilitators felt that LBT was similarly beneficial for neurotypical children and young people. Others reported that the benefits seen for autistic children and young people did not always translate to environments outside of the group therapy session. Many facilitators noted that they will continue to use the skills gained from delivering LBT both inside and outside the classroom, for example, LBT had given them another tool to help autistic children struggling with communication.

##### Self‐efficacy

Most facilitators felt that the training they received as part of the I‐SOCIALISE study adequately prepared them to deliver LBT. However, a small number stated they would have liked further training on conflict resolution between group members. Some also noted the benefits of group training, as they were able to ‘bounce ideas’ off each other.

I would say, you know, maybe in the training, go over a little bit more the issues you may encounter and how to deal with those. (Primary facilitator)

Nearly all facilitators agreed that TAs have the appropriate skills to be able to deliver LBT. Most suggested that patience is the most important skill, especially when facilitating problem solving between participants. Facilitators also felt that group dynamics were a key factor for LBT to be successful, and knowledge of relationships between the participants will make delivering the programme easier.

I think they need to be very patient and understanding. And I think they need to know their children and know what makes them tick. (Primary facilitator)

## DISCUSSION

The aim of this embedded mixed‐methods study was to examine the acceptability of LBT delivered in mainstream primary and secondary school environments for facilitators, and parents/guardians on behalf of their children and young people. The findings suggest that LBT is acceptable to facilitators and parents/guardians.

Facilitators reported that overall, children and young people and wider school staff viewed the programme positively. Facilitators also reported observing improvements in communication and social skills for autistic children and young people during the LBT sessions, but the findings were less clear about the benefits in other school settings. Some facilitators described improvements, such as socializing in the playground, whilst others failed to see changes outside of the sessions. Parents/guardians also rated the programme positively, but had mixed views on whether improvements had been seen at home. Organizing LBT in school settings requires careful planning, including issues with resources and staff schedules, but facilitators reported that these challenges did not outweigh the benefits. Overall LBT was perceived as a worthwhile programme.

Similar findings of the acceptability of this programme to children and young people has been reported elsewhere including them rating enjoyment of the sessions very highly (Evans et al., 2012; Owens et al., 2008) and enjoying aspects such as freestyle LEGO play (Brett, 2013). Teachers in previous research have also reported that therapy using LEGO is effective at improving social skills as well as being appealing, enjoyable and motivating (Griffiths, 2016). Older research into social skills interventions showed that acceptability ratings by teachers (often delivered face to face by adult professionals) showed variability in acceptability depending on the techniques being used (Odom et al., 1993). This may be related to the previous tendency to design interventions around perceived ‘deficits’ and didactically try and teach adaptive social behavior (see examples in the systematic reviews by Gates et al., 2017; Wolstencroft et al., 2018). In recent years interventions tend to be more child centered for example by paying closer attention to child individual and cultural needs (Davenport et al., 2018) or using interventions that are of interest to children and young people with ASD such as technologically based or fun interventions (Mosher et al., 2021). Worryingly, acceptability is often not considered in research in this field (see systematic reviews by Gates et al., 2017; McCoy et al., 2016; Wolstencroft et al., 2018). This may be because child rated acceptability can be very complex to obtain accurately, but it remains important information given that helping children and young people with ASD with their social relationships in positive ways may improve engagement and importantly their mood (Rumney & MacMahon, 2017).

The strengths of this study lie in the high completion rates for the questionnaires completed by facilitators and parents/guardians (93% and 77%, respectively). The use of Sekhon et al.'s (2017) Theoretical Framework of Acceptability to inform the design and structure of data collection and analysis was helpful in unifying understanding of a broad concept of acceptability and exploring acceptability using two different approaches. This approach will also enable the comparison to studies with similar interventions that go on to use the TFA in the future. A further strength of this research was the participation of teaching staff and parent representatives with experience of LBT in the design and trialing of both the questionnaire and interview schedule.

### Limitations

Several limitations of this study have been identified. Firstly, the study only included mainstream schools therefore further research would be necessary to explore the effectiveness in special educational needs schools. Secondly, the study did not collect data directly from the children and young people who participated in the programme. The original proposal for this study included planned qualitative interviews with parents and participants in the intervention arm but, prior to confirmation of funding, the team were asked to reduce the scope of the acceptability study to interviews with school staff only. Although both facilitators and parents/guardians reflected on the perceived experiences and opinions of the children and young people, it is likely that a deeper insight into the acceptability of LBT to children and young people would have been gained by inviting children and young people to complete a feedback questionnaire or interview directly.

A further limitation may have been that only facilitators who found LBT to be acceptable took part in the interviews and the data may reflect this. To address this, we extended the sample size from the planned 12 to 16 in a bid to capture greater diversity in the views on facilitators. Data collection ceased at 16 interviews as we reached saturation. Finally, a greater number of facilitators from secondary schools would have been beneficial to allow the exploration of acceptability of LBT in a secondary school setting to be explored and compared to a primary setting even further. Despite these limitations, the survey and interview data consistently portray LBT positively, suggesting the limitations may not have had a large impact.

The high degree of acceptability of this programme in this study is encouraging and supports the wider implementation of the programme if (i) the evidence on effectiveness and cost‐effectiveness supports adoption; (ii) the funding for training and resources is identified; (iii) decisions are made to include the programme as part of the mainstream school‐based provision for children and young people with ASD and (iv) appropriate resources to support delivery of the programme are available to schools. We know that autistic children and young people are not asocial but socialize in different ways to neurotypical peers (Wright et al., 2020) and that they can experience loneliness (Baczewski & Kasari, 2021). These aspects of social isolation are important when considering interventions that not only bring children and young people with ASD into contact with other children around a common and enjoyable interest but enhance and nurture skills that can improve their social world and reduce isolation.

## CONCLUSION

We found that LBT had a high degree of acceptability reported by facilitators and parents/guardians, however, facilitators rated the acceptability of the programme higher overall than parents. Given the increasing emphasis on the role of schools in seeking to improve social outcomes for children and young people with ASD, our study shows that LBT may be an attractive and acceptable options for schools.

## CONFLICT OF INTEREST

Gina Gomez de la Cuesta co‐authored the LEGO®‐based therapy manual which formed the basis of the intervention delivered in the trial. The co‐authors of the manual have given us full permission to use the manual without license and to develop an abridged version. They have also stated their support for us in writing our own version and will become co‐authors on any future publications. Co‐applicant Gomez de la Cuesta has also agreed for the team to adapt the fidelity checklist used in her previous study. Co‐applicant Gomez de la Cuesta is a Director of Play Included a community interest company that offers training and resources for interventions involving play bricks for children. All other authors have no conflicts of interest to declare.

## Supporting information


**Supplementary information 1** Inclusion and exclusion criteria for the I‐SOCIALISE trial (Varley et al., 2019)

Appendix S2

Appendix S3

Appendix S4

**Supplementary information 5** Summary of coding framework

**Supplementary information 6** Acceptability of LEGO®‐based therapy mapped to Theoretical Framework of Acceptability (TFA) constructs

## Data Availability

Requests for patient level data and statistical code should be made to the corresponding author and will be considered by members of the original trial management group, including the chief investigator and members of CTRU, who will release data on a case‐by‐case basis. The data will not contain any direct identifiers, we will minimise indirect identifiers and remove free text data, to minimise the risk of identification.
