# Supplementary material for: A mixed methods evaluation of the acceptability of therapy using LEGO® bricks (LEGO® based therapy) in mainstream primary and secondary education
Source: Autism Res. 2022 Apr 9;15(7):1237–48. doi: 10.1002/aur.2725 (PMC9324108; doi:10.1002/aur.2725)
Supplement: Supplementary file 1 — Supplementary information 1 Inclusion and exclusion criteria for the I‐SOCIALISE trial (Varley et al., 2019) [file AUR-15-1237-s001.docx]

**Supplementary information 1: Inclusion and exclusion criteria for the I-SOCIALISE trial (Varley et al., 2019)**

**Inclusion criteria:**

A school will be included if:

- It is a mainstream school in ethically approved participating localities.
- It has not used LEGO-based therapy with the child in the current or preceding school term.
- They have at least one child diagnosed with ASD (in line with child inclusion criteria below).

A child will be included if:

- They are aged between 7 and 15 years and attend a mainstream school in years 2–10.
- The child and parent/guardian have a sufficient understanding of English to be able to provide informed assent/consent and read the LEGO-based therapy instructions.
- They have an ASD clinical diagnosis from a qualified assessing clinician or team (based on best-practice guidance leading to the 10th revision of the International Statistical Classification of Diseases and Related Health Problems (World Health Organisation, 1993) or Diagnostic and Statistical Manual of Mental Disorders, Fifth Edition (American Psychiatric Association, 2000) diagnosis as reported by the child’s parent/guardian and in the child’s school records).
- They score 15 or higher on the Social Communication Questionnaire (SCQ).
- They have the ability to follow and understand simple instructions.

**Exclusion criteria**

- The child has a physical impairment which would prevent them participating in the activities.

1. World Health Organization. The ICD-10 classification of mental and

behavioural disorders: Diagnostic criteria for research. Geneva: World Health Organization, 1993.

1. American Psychiatric Association. Diagnostic and statistical manual of mental disorders: DSM-IV-TR. 4th edn, text revision. Washington, DC: American Psychiatric Association, 2000.
