# Supplementary material for: A mixed methods evaluation of the acceptability of therapy using LEGO® bricks (LEGO® based therapy) in mainstream primary and secondary education
Source: Autism Res. 2022 Apr 9;15(7):1237–48. doi: 10.1002/aur.2725 (PMC9324108; doi:10.1002/aur.2725)
Supplement: Supplementary file 2 — Appendix S2 [file AUR-15-1237-s005.pdf]

Parent acceptability questionnaire

Region / School / Participant

Child's initials

We are keen to get your feedback on the LEGO®-based therapy that your child has received as part of the I-SOCIALISE study. For each of the statements below, please tick the box that best represents your views.

		Strongly agree	Agree	Neither agree nor disagree	Disagree	Strongly disagree
**1.**	LEGO®-based therapy fits in well with my views on what helps children with autism	☐	☐	☐	☐	☐
**2.**	Taking part in LEGO®-based therapy was a negative experience for my child	☐	☐	☐	☐	☐
**3.**	Taking part in LEGO®-based therapy was a big effort for my child	☐	☐	☐	☐	☐
**4.**	Taking part in LEGO®-based therapy was not time well spent instead of doing other activities that might help my child	☐	☐	☐	☐	☐
**5.**	LEGO®-based therapy made a positive impact on my child's *social skills*	☐	☐	☐	☐	☐
**6.**	LEGO®-based therapy made a negative impact on my child's *academic confidence*	☐	☐	☐	☐	☐
**7.**	LEGO®-based therapy made a negative impact on my child's *communication skills*	☐	☐	☐	☐	☐
**8.**	LEGO®-based therapy made a positive impact on my child's *behaviour*	☐	☐	☐	☐	☐
**9.**	My child does not have a clear understanding of what LEGO®-based therapy is	☐	☐	☐	☐	☐
**10.**	My child felt confident about how to take part in LEGO®-based therapy	☐	☐	☐	☐	☐
**11.**	LEGO®-based therapy has helped my child	☐	☐	☐	☐	☐

Additional comments – please use this space to provide any other feedback on LEGO®-based therapy
