# Supplementary material for: A mixed methods evaluation of the acceptability of therapy using LEGO® bricks (LEGO® based therapy) in mainstream primary and secondary education
Source: Autism Res. 2022 Apr 9;15(7):1237–48. doi: 10.1002/aur.2725 (PMC9324108; doi:10.1002/aur.2725)
Supplement: Supplementary file 3 — Appendix S3 [file AUR-15-1237-s002.pdf]

Interventionist acceptability questionnaire

Region / School / Interventionist: ☐ / ☐☐ / I ☐

Interventionist's initials ☐☐☐

We are keen to get your feedback on LEGO®-based therapy. For each of the statements below, please tick the box that best represents your views.

Date of completion ☐☐ ☐☐ ☐☐☐☐
d d m m y y y y

		Strongly agree	Agree	Neither agree nor disagree	Disagree	Strongly disagree
1.	LEGO®-based therapy fits in well with my views on what helps children with autism	☐	☐	☐	☐	☐
2.	Delivering LEGO®-based therapy was a negative experience for me	☐	☐	☐	☐	☐
3.	Delivering LEGO®-based therapy was a big effort for me	☐	☐	☐	☐	☐
4.	Delivering LEGO®-based therapy was not time well spent instead of doing other activities to help children with autism	☐	☐	☐	☐	☐
5.	LEGO®-based therapy made a positive impact on the child(ren)'s *social skills*	☐	☐	☐	☐	☐
6.	LEGO®-based therapy made a negative impact on the child(ren)'s *academic confidence*	☐	☐	☐	☐	☐
7.	LEGO®-based therapy made a negative impact on the child(ren)'s *communication skills*	☐	☐	☐	☐	☐
8.	LEGO®-based therapy made a positive impact on the child(ren)'s *behaviour*	☐	☐	☐	☐	☐
9.	I do not have a clear understanding of what LEGO®-based therapy is	☐	☐	☐	☐	☐
10.	I felt confident in delivering LEGO®-based therapy	☐	☐	☐	☐	☐
11.	LEGO®-based therapy has helped the child(ren) with autism	☐	☐	☐	☐	☐

Additional comments – please use this space to provide any other feedback on LEGO®-based therapy
