# Supplementary material for: A mixed methods evaluation of the acceptability of therapy using LEGO® bricks (LEGO® based therapy) in mainstream primary and secondary education
Source: Autism Res. 2022 Apr 9;15(7):1237–48. doi: 10.1002/aur.2725 (PMC9324108; doi:10.1002/aur.2725)
Supplement: Supplementary file 4 — Appendix S4 [file AUR-15-1237-s006.docx]

**I-SOCIALISE: Interview schedule for interventionist interviews**

**Introduction**

- Introduce yourself and explain that the purpose of the interview is get their feedback on delivering Lego®-based therapy within their school.
- Before the interview begins, check whether the participant has any questions about the interview, check that they are happy to go ahead with the interview (re-establish verbal consent) and agree to the interview being recorded.

***1. Coherence (meaning and sense-making)***

1. What is easy or difficult to describe about Lego®-based therapy when you’re talking to children, parents or other teaching staff?
2. What makes Lego®-based therapy different from other interventions for children with autism?
3. Does it have a clear purpose that is shared between children, parents and other teaching staff?
4. What benefits do you think Lego®-based therapy will bring, and to whom?
5. How are these benefits likely to be valued?
6. How does Lego®-based therapy fit with the overall goals and activity of your school?
7. How does Lego®-based therapy fit with your own value system?

- How does this fit with your views on what helps children with autism?

***2. Cognitive participation (commitment and engagement)***

1. What are children, parents and your colleagues’ views of Lego®-based therapy?
2. Do they think it’s a good idea?
3. Do they see the point of the intervention?
4. Were children, parents and teaching staff prepared to invest time, energy and work in Lego®-based therapy?

- What are the opportunity costs of Lego®-based therapy?
- Are the likely benefits of Lego®-based therapy worth the time, energy and work that needs to be put in?

***3. Collective action (the work teaching staff and children do to make the intervention work)***

1. How has delivering Lego®-based therapy affected your work?

- In what ways has this promoted or impeded your work?
- What effect has it had on how you work with children with autism?

1. How compatible is Lego®-based therapy with existing work practices?
2. Will teaching staff require extensive training before they can deliver Lego®-based therapy?
3. Do all teaching staff working with children with autism have the right set of skills to deliver Lego®-based therapy?
4. Did you feel that the training you received prepared you adequately to deliver Lego®-based therapy as part of the I-SOCIALISE study?

- Did you feel confident to deliver the intervention?

1. What impact does Lego®-based therapy have on:

- The division of labour in your school
- Resources
- Responsibility between different professional groups

1. Who has the power to make Lego®-based therapy happen in your school?

***4. Reflexive monitoring (professionals reflect on or appraise the intervention)***

1. How are children, parents and teaching staff likely to perceive Lego®-based therapy once it’s been ongoing for a while?
2. Is it likely to be perceived as advantageous for children, parents or staff?
3. What effects has Lego®-based therapy had?
4. What effects do you think may still occur?
5. Have you received any feedback from teaching staff, children or parents about Lego®-based therapy?
6. How would you adapt or improve the intervention based on experience?

Is there anything else that you would like to say about Lego®-based therapy?
