# Supplementary material for: A mixed methods evaluation of the acceptability of therapy using LEGO® bricks (LEGO® based therapy) in mainstream primary and secondary education
Source: Autism Res. 2022 Apr 9;15(7):1237–48. doi: 10.1002/aur.2725 (PMC9324108; doi:10.1002/aur.2725)
Supplement: Supplementary file 5 — Supplementary information 5 Summary of coding framework [file AUR-15-1237-s003.docx]

**Supplementary information 5: Summary of coding framework**

| **Category 1. Understanding** | | | |
| --- | --- | --- | --- |
| Code 1.1 Parent | | | |
| 1.1.1 Terminology | | 1.1.2 More than playing | |
| 1.1.3 Observation | | 1.1.4 Explaining | |
| Code 1.2 Teacher | | | |
| 1.2.1 Terminology | | 1.2.2 More than playing | |
| 1.2.3 Observation | | 1.2.4 Explaining | |
| Code 1.3 Child | | | |
| 1.3.1 Terminology | | 1.3.2 More than playing | |
| 1.3.3 Observation | | 1.3.4 Explaining | |
| Code 1.4 Intervention differences | | | |
| **Category 2. Benefits** | | | |
| Code 2.1 CYP with ASD | | | |
| 2.1.1 Communication | 2.1.2 Socialising | | 2.1.3 Confidence |
| 2.1.4 Language Skills | 2.1.5 Concentration | | 2.1.6 Patience/calmness |
| 2.1.7 Wider benefits | 2.1.8 Resilience | | 2.1.9 Fine motor skills |
| Code 2.2 CYP without ASD | | | |
| 2.2.1 Communication | 2.2.2 Socialising | | 2.2.3 Confidence |
| 2.2.4 Language Skills | 2.2.5 Concentration | | 2.2.6 Patience/calmness |
| 2.2.7 Wider benefits | 2.2.8 Resilience | | 2.2.9 Fine motor skills |
| Code 2.3 Professional | | | |
| **Category 3. Implementation** | | | |
| 3.1 Continued therapy | 3.2 Expanded to other CYP | | 3.3 School/staff support |
| 3.4 Commitment | 3.5 Integration into practice | | 3.6 Cost of LEGO® |
| **Category 4. Values** | | | |
| 4.1 School values | | 5.2 Professional values | |
| **Category 5. Resources** | | | |
| 5.1 Opportunity costs | 5.2 Staffing | | 5.3 Space |
| 5.4 Time | 5.5 Knowledge of ASD | |  |
| **Category 6. Working practices** | | | |
| 6.1 Power | 6.2 Training | | 6.3 Adaptations |
| 6.4 Interventionist skills | 6.5 Recommendations | | 6.6 Importance of relationships |
| **Category 7. Acceptability** | | | |
| 7.1 Enjoyment of LEGO® | | 7.2 Examples | |
| **Category 8. Challenges**^†^ | | | |
