# Supplementary material for: A mixed methods evaluation of the acceptability of therapy using LEGO® bricks (LEGO® based therapy) in mainstream primary and secondary education
Source: Autism Res. 2022 Apr 9;15(7):1237–48. doi: 10.1002/aur.2725 (PMC9324108; doi:10.1002/aur.2725)
Supplement: Supplementary file 6 — Supplementary information 6 Acceptability of LEGO®‐based therapy mapped to Theoretical Framework of Acceptability (TFA) constructs [file AUR-15-1237-s004.docx]

**Supplementary information 6: Acceptability of LEGO®-based therapy mapped to Theoretical Framework of Acceptability (TFA) constructs**

| **TFA construct** | **Category(s)** |
| --- | --- |
|  |  |
| Affective attitude | 1. Understanding  7. Acceptability  8. Challenges |
| Burden | 3. Implementation  5. Resources |
| Ethicality | 4. Values |
| Intervention coherence | 1. Understanding |
| Opportunity costs | 4. Values |
| Perceived effectiveness | 2. Benefits  8. Challenges |
| Self-efficacy | 6. Working practices |

|  | **TFA constructs** | | | | | | |
| --- | --- | --- | --- | --- | --- | --- | --- |
| **Category** | Affective attitude | Burden | Ethicality | Intervention coherence | Opportunity costs | Perceived effectiveness | Self-efficacy |
| 1. Understanding | X |  |  | X |  |  |  |
| 2. Benefits |  |  |  |  |  | X |  |
| 3. Implementation |  | X |  |  |  |  |  |
| 4. Values |  |  | X |  | X |  |  |
| 5. Resources |  | X |  |  |  |  |  |
| 6. Working practices |  |  |  |  |  |  | X |
| 7. Acceptability | X |  |  |  |  |  |  |
| 8. Challenges | X |  |  |  |  | X |  |
